# An improved method of crafting a multi-electrode spiral cuff for the selective stimulation of peripheral nerves

**DOI:** 10.1038/s41598-018-19318-w

**Published:** 2018-01-17

**Authors:** Janez Rozman, Polona Pečlin, Samo Ribarič, Matjaž Godec, Jaka Burja

**Affiliations:** 1 Center for Implantable Technology and Sensors, ITIS d. o. o. Ljubljana, Lepi pot 11, 1000 Ljubljana, Slovenia; 2 Institute of Pathophysiology, Medical Faculty, University of Ljubljana, Vrazov trg 2, 1000 Ljubljana, Slovenia; 3 Institute of Metals and Technology, Lepi pot 11, 1000 Ljubljana, Slovenia

## Abstract

This article reviews an improved methodology and technology for crafting a multi-electrode spiral cuff for the selective activation of nerve fibres in particular superficial regions of a peripheral nerve. The analysis, structural and mechanical properties of the spot welds used for the interconnections between the stimulating electrodes and stainless-steel lead wires are presented. The cuff consisted of 33 platinum electrodes embedded within a self-curling 17-mm-long silicone spiral sheet with a nominal internal diameter of 2.5 mm. The weld was analyzed using scanning electron microscopy and nanohardness tests, while the interconnection was investigated using destructive load tests. The functionality of the cuff was tested in an isolated porcine vagus nerve. The results of the scanning electron microscopy show good alloying and none of the typical welding defects that occur between the wire and the platinum foil. The results of the destructive load tests show that the breaking loads were between 3.22 and 5 N. The results of the nanohardness testing show that the hardness of the weld was different for the particular sites on the weld sample. Finally, the results of the functional testing show that for different stimulation intensities both the compound action potential deflection and the shape are modulated.

## Introduction

Many biomedical devices that interact with the peripheral nervous system have been developed for both recording and stimulation. Among a number of factors, the functionality of stimulating electrode systems, implanted onto peripheral nerves in humans, depend on the design, motion, mechanics, material composition and surface properties. All applications of chronically implanted neural prostheses require electrodes that are capable of providing a safe and reliable electrical stimulation that is dependent on the surface and bulk properties of the used materials. Material-modification techniques that optimize both the surface and bulk properties should be applied. Because the installation of such an electrode system for a peripheral nerve potentially causes mechanical injuries, it is necessary to consider and resolve all these issues.

Considerable scientific and technological efforts have been devoted to developing systems of electrodes that interface the human nervous system with implantable electronic devices. Recent work has also led to advances in cuffs that enable selective fibre-bundle functional electrical stimulation^1^. As a result, multi-electrode nerve cuffs have been used for the electrical stimulation of peripheral nerves for several decades^2–4^. Twenty years ago, Haugland^5^ presented a simple method for constructing nerve cuffs based on using platinum foil electrodes and silicone. *In-vivo* testing showed that in a 1-year period none of the cuffs failed. However, the cuffs were designed to have a small number of circular electrodes enabling only whole-nerve stimulation instead of numerous individual electrodes to stimulate selectively particular superficial compartments. The trend in neural prostheses that use selective nerve stimulation for therapy is towards single-part systems with a large number of stimulating electrodes, each of which selectively stimulates neural tissue or records a neural response.

Among the different multi-electrode designs, the self-sizing spiral design was chosen for the cuff, because of its ability to selectively stimulate superficial compartments of the nerve when wrapped around^6–10^. Spiral cuffs were used to re-establish the diminished function of internal organs and glands such as the heart, lungs, stomach, bladder and pancreas or to treat chronic diseases in humans by stimulation of the vagus nerve^11–17^. Advances in vagus-nerve stimulation have increased the need for cuff designs that can selectively modulate the function of the desired internal organ also via the selective stimulation of selected populations of nerve fibres. This selectivity has proved to be difficult to achieve^9,18^. As a result, most cuffs are manufactured using medical-grade silicone materials in combination with platinum foils that are connected to stainless-steel lead wires using different technologies.

Although cuffs are not entirely without disadvantages, the mechanical interface, and thus the reproducibility of selective stimulations, is still superior to alternative solutions. External pressures applied to the peripheral nerve can elicit blood flow occlusion that leads to nerve degeneration and demyelination. To ensure the highest possible biocompatibility and stability, when implementing a cuff for nerve stimulation, this tissue damage must be avoided. Therefore, the technology for crafting the spiral nerve cuffs should consider cuff-tissue interactions as well as the appropriate technological solution. Grill and Mortimer^19^ reported on the input-output properties of chronically implanted, silicone spiral cuffs, containing 12 platinum electrodes implanted on the sciatic nerves of cats. Their results suggested that tissue encapsulation actually stabilized the chronically implanted cuffs, while in more than half of the cuffs, the interconnections and/or lead wires were irreparably damaged. Racz and Heavner^20^ suggested that the use of the cuff for peripheral nerve stimulation has little future due to the fact that the cuff does not allow the stimulation of individual axons. However, advances in cuff design has enabled systems with a number of electrodes that are chronically interfaced to the peripheral autonomous nervous system^6,21,22^.

Among the different materials used to produce stimulating electrodes, platinum is commonly used in the form of a pure metal (99.93 wt %). The relatively low strength of pure platinum can be increased in multi-electrode stimulating systems by plastic deformation and partial annealing. To improve the mechanical properties of platinum, to analyse any failure and determine its microstructure, metallographic analysis with electrolytic etching can be used^23,24^. Apart from chemical inertness, platinum also has a number of physical properties of great value for use in implantable stimulating electrode technology. The structural properties of a cold-rolled platinum foil used to manufacture multi-electrode spiral nerve cuffs that can be additionally improved with thermal treatments were considered in the study^25,26^.

For crafting a cuff, resistance spot welding was proposed. It is a process in which the contacting metal surfaces are joined using the heat obtained from the resistance to electric current. The standard process uses two shaped electrodes to clamp the sheets together and to concentrate the large welding current into a small spot, thus melting the metal and forming the weld^27–29^. Capacitive discharge spot welding provides high peak currents with extremely fast pulse rise times (10 s of µs). Thus welds occur quickly enough for the spots to be small and have very small heat-affected zones. This is very important for two reasons. The first is the need for the electrochemical properties of the affected zone and weld to remain confined within the safe region, since the electrolyte’s composition adjacent to the electrode and the finite rate of faradaic reactions can lead to irreversible processes that cause significant damage to the electrode or tissue injury. The second one is the stiffness of the cuff that is closely related to the procedures of crafting, where stimulating electrodes are mounted on a substrate made of medical-grade silicone, while the lead wires are spot welded onto the electrodes.

The objectives of the study are to (a) investigate the feasibility of spot welding the interconnections between platinum stimulating electrodes and strands of stainless-steel lead wires within the cuff, (b) define the mechanical interface between the platinum stimulating electrode and the lead wires that influences its neurophysiological performance and (c) develop a cuff capable of reshaping the nerve geometry slowly and controllably, applying a low value of radial pressure to the nerve with diameters ranging from 2 to 4 mm^30^. A tight positioning of the cuff on the nerve enables the highest-possible degree of extra-neural selective activation of the nervous tissue. Also, the threshold stimulus currents are low, the activating function is great, and the power requirements for the stimulator are low. It is also very important that the cuff should provide stable recruitment patterns over time, and the opportunity for an as-simple-as-possible retrieval and replacement^31^. The ultimate aim was to contribute to the further development of a cuff for an efficient, safe and selective nerve stimulation that would advance the further development of neural prostheses in humans.

## Materials and Methods

### Development of models

Nerve cuffs have been successfully used for stimulating peripheral nerves^32–35^. However, before any successful use in chronic, long-term, selective vagal nerve stimulation, additional tests regarding the potential induction of functional and morphological changes following chronic implantation have to be made^36–39^. Besides this, an entire and appropriate physical model, including technical limitations, was considered.

The design of the cuff was based on the realistic structural topography of a porcine cervical vagus nerve and the published models of activation for different types of nerve fibres within a specific superficial region of the nerve^7,40–50^. The functionality paradigm of the cuff was tested recently intra-operatively in humans, where the vagus nerves were stimulated selectively to modulate the function of the heart as well as in “*ex vivo*” experiments where the left porcine vagus nerves were selectively stimulated under simulated physiological conditions^51,52^. In the latter, the paradigm for spatial and fibre-type selective vagus nerve stimulation was developed using the realistic structural topography of a porcine cervical left vagus nerve.

**Figure 1:**
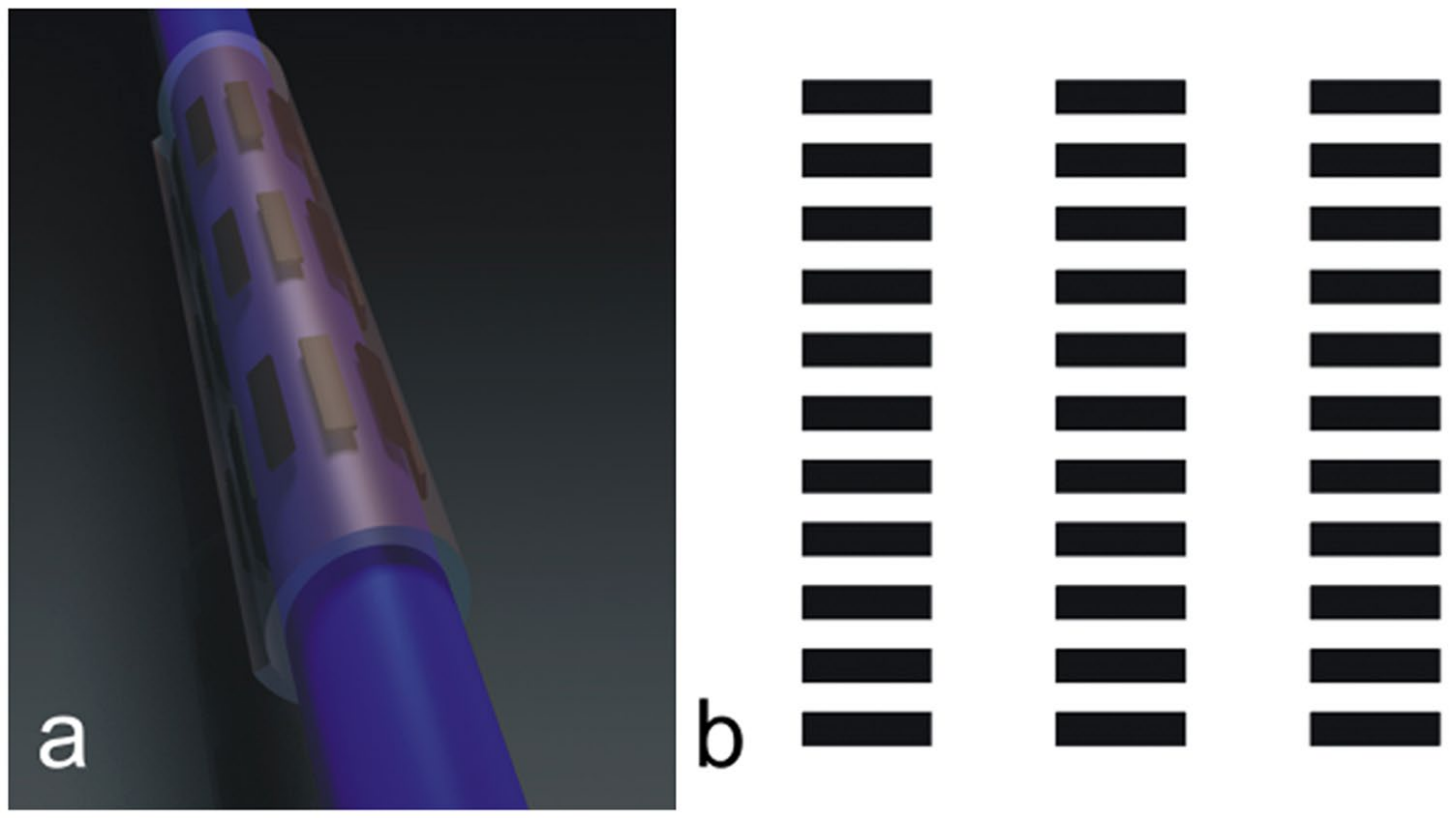
(**a**) 3D illustration of the stimulating spiral nerve cuff. (**b**) Integrated matrix of 33 electrodes.

As a result of the design, the electrodes were arranged in a spirally rolled matrix of three parallel groups, each containing thirteen electrodes and forming thirteen longitudinal groups of three electrodes. Each stimulation electrode embedded in the cuff is connected to an insulated lead wire. The specifications of the cuff that resulted from the modelling are as follows: nominal nerve diameter (inner diameter of the first layer), 2.5 mm; cuff length, 17 mm; turns in resting position, 1.5; electrode length, 2.5 mm; electrode width, 0.7 mm; circumferential separation between electrodes, 0.5 mm; and longitudinal separation between electrodes, 2.5 mm. The modelled thirty-three-electrode cuff is shown schematically in Fig. 1.

### Selection of materials

To minimize the possible mechanical damage and fitness difficulties in the application of cuffs, flexible substrates and metallic materials with the appropriate mechanical characteristics were selected^53^.

Other criteria that were considered when choosing the material for the electrodes that make electrical contact with the neural tissue were the mechanical characteristics of the material. In the electrical stimulation of nerves, platinum is commonly used as the stimulating electrode material because it can effectively supply high-density electrical charge to activate the neural tissue predominantly by capacitive and faradaic mechanisms^54–56^. However, in the selective nerve stimulation with a higher charge density, metallic dissolution products, hydrogen and oxygen gas bubbles, oxidized organic and inorganic species, and pH shifts causing irreversible changes in the tissue proteins could occur^53,57^.

### Fabrication of the platinum foil

Since the mechanical characteristics, the working characteristics and the stability against corrosion in physiological media strongly depend on the impurity content, a purity of at least 99.9 wt % is required for the stimulating electrodes. Pt also has many physical properties that are of great value for their use in the technology of implantable stimulating electrodes. These include general properties, mechanical properties, and physical properties. One of them is mechanical fitness, which is an important issue in the design of multi-electrode stimulation systems^58^. In this regard, the low strength of the high-purity platinum (99.93 wt %) has been accepted in the stimulating electrodes. However, in order to optimise the technical parameters for the thermal and mechanical processing of platinum, its rheological characteristics, including its deformation resistance, were characterized^59,60^. The specific properties of the platinum that were considered when the platinum foil was fabricated are given in Table 1.

**Table 1:** Mechanical properties of platinum.

Condition	Tensile strength (MPa	Elongation (%)	Hardness Vickers (HV)
As-worked	207–241	1–3	90–95
Annealed	124–165	30–40	/

In spite of the low strength of pure platinum, stimulating electrodes with greater strength can be made using optimum working cycles of the plastic deformation and partial appropriate annealing regimes determined for the microstructure of the platinum^23,24,61,62^.

Therefore, in the development of the cuff, superficial and structural properties of a cold-rolled platinum foil used to craft stimulating electrodes were considered. Nevertheless, the long-term electrochemical stability of the stimulating electrodes presumably also depends on the long-term stability of the platinum crystal grains^63^.

The platinum foil is obtained from a platinum ingot of chemical composition 99.99 wt %, Rh 0.01 wt% by cold forging into a rectangular rod (11 mm × 11 mm) and cold rolling into a ribbon with a thickness of 8 mm. The obtained platinum ribbon, however, experienced a considerable strain hardening during cold rolling. To renew the softness and ductility of the ribbon, re-crystallization was promoted by subsequent annealing at 1040 °C for 5 minutes and cooling in water at 20 °C. Further reductions in the thickness of the ribbon obtained by cold rolling are as follows: 5.0 mm, 2.5 mm, 1.2 mm, 0.6 mm, 0.3 mm, 0.16 mm, and 0.06 mm. After each reduction the softness and ductility of the ribbon were promoted by subsequent annealing at 1040 °C for 5 minutes and cooling in water at 20 °C. The last reduction obtained by cold rolling resulted in the foil having narrow tolerances with a final thickness of 0.03 mm. After the last reduction, however, the foil was not thermally treated and remained in the hardened state.

### Structural inspection of the platinum foil

The results and information about the temperature-dependent electrical resistivity, the nano-indentation measurements, the differential scanning calorimetry (DSC) and the optical microscopy of a platinum foil treated using cold rolling and annealing at different temperatures shown in the literature and from our previous studies were considered^61–63^.

### Development of the nerve cuff

The silicone spiral cuff was crafted using non-reinforced, medical-grade silicone sheet with a thicknesses of 0.05 mm (0.002″), which is a highly flexible silicone elastomer. The medical-grade silicone sheeting is chosen because of its several advantages, including: a high degree of chemical inertness and bio-compatibility, ease of cutting to size and shape, and a high permeability to gases.

The cuff was crafted by bonding the two aforementioned 0.05-mm-thick silicone sheets together. One sheet, stretched and fixed in position, was covered with a thin layer of adhesive. The second, un-stretched sheet was placed on top of the adhesive, and the composite was compressed to a thickness of approximately 0.15 mm. When released after curing, the composite curled into a spiral tube as the stretched sheet contracted to its natural length^1,11,18,51^.

### Crafting of the stimulating electrodes

The crafting of the stimulating electrodes involves several handcrafting steps. The first one is cutting the 0.7-mm-wide strips out of the 0.03-mm-thick platinum foil using a sharp blade. The second one is cutting 7.5-mm-long individual strips. The third one is folding the strips at one- and at two-thirds to obtain U-shaped forms. Afterwards, thirty-three rectangular electrodes were mounted on a third silicone sheet.

Since superior quality, a high flexibility, a long flex-life and miniaturization are required for the custom cable assembly of the cuff, stranded stainless-steel wire of type AS 631 from Cooner Wire, Chatsworth, CA, USA was used (the specifications are given in Table 2). Each of the multi-conductor wires is constructed from finely stranded stainless-steel wire and extruded thin-wall insulation made of fluorinated ethylene propylene (FEP).

**Table 2:** Specifications of the stranded stainless-steel multi-conductor cable AS 631.

Wire Series	AWG Size	Conductor Circ. Mils	Strand Constr.	Nom. Wall Thickness FEP	Nominal Overall Diameter	OHMS Per Foot
AS 631	40	10	10/50	0.003–0.004	0.011	45.94

Type 316 L austenitic stainless steels are molybdenum-bearing (2 to 3%) steels that are resistant to general corrosion and pitting/crevice corrosion. In addition to excellent corrosion resistance, the 316 L steel grade also provides excellent fabrication and formability. Austenitic stainless steels are considered to be the most weldable of the stainless steels. They are routinely joined by fusion- and resistance-welding processes.

Professional micro spot-welders such as Powerstream, MTI Microwelding, Spotco, MacGregor, etc., are designed to cover a wide range of applications and are usually expensive. Since the manufacture of this kind of stimulating multi-electrode system is a very precise and time-consuming process, we do not need the precise pulse shaping offered by high-end professional devices, but instead we require a highly experienced and well-trained operator.

**Figure 2:**
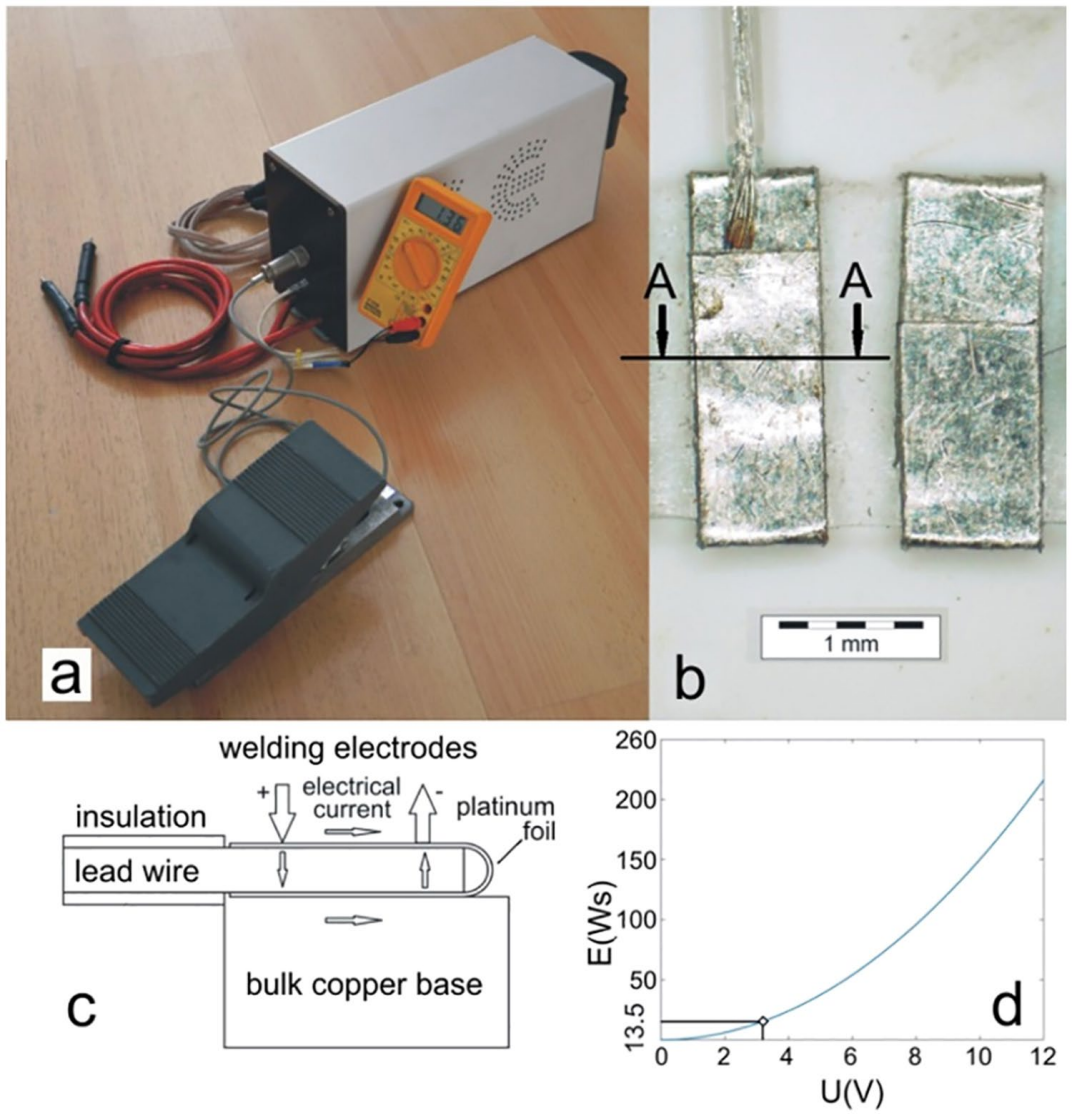
(**a**) Micro spot-welding device; (**b**) Welded interconnection with indicated cross-section A-A for metallographic analysis; (**c**) Schematic diagram of welding an interconnection between the stimulating electrodes and the lead wire; (**d**) Welding energy versus charging voltage, showing the region where optimum and reproducible results are obtained.

For the purpose of cuff crafting using high-quality micro spot welds, an inexpensive capacitive discharge research spot welder shown in Fig. 2a providing a standard single-pulse was developed. For this purpose, a custom design based on a thyristor powered by a large capacitor discharge was deployed. We dumped all the capacitor energy at once via a thyristor. Therefore, to obtain good and reproducible results, we must be careful that the input energy is not too small so that the joint is robust enough and that we do not to burn a hole through the material. To match the sheet’s material properties, its thickness, and the type of electrodes, the amount of energy delivered to produce repeatable fusion welds was chosen on a subjective basis by the operator. The used capacitor of 3 farads was able to produce a voltage as high as 35 V, and thus able to store up to 1837.5 W of energy available for heavy-duty work. Since for the proposed application of welding the lead wire was sandwiched between the two ends of a bent platinum ribbon, the available energy to obtain good and reproducible results was limited to a maximum of 216 W. The energy stored within the voltage range between zero and 12 DCV and calculated according to equation (1) is shown in Fig. 2d. The figure also shows the region where the optimum welding energy and reproducible results were obtained.

1 $${{\rm{U}}}_{{\rm{E}}}={\rm{C}}\cdot {{\rm{U}}}^{{\rm{2}}}/2$$

In this regard, the properties of the platinum and the 316 L stainless steel relevant for establishing the optimum welding conditions were the melting point and the electrical resistivity that were 1769 °C and 10.6 μΩ·cm at 25 °C for the platinum and 1390 to 1440 °C and 69 μΩ·cm at 25 °C for the stainless steel.

Two penholder-like handles of the spot welder were made from 12-mm-diameter rods made of very pure copper, while commercially available 3-mm-diameter tungsten welding electrodes shaped specifically at the outer end were inserted into each of the handles and tightened with an M8 nut. The handles are also insulated using heat-shrink tubing to avoid electrical short circuits. The number on the display of the instrument shows the capacitor voltage that can be selected using dials on the power supply. After triggering with a foot-switch pedal, the actual pulse energy stored in the capacitor is dumped via a thyristor onto the sandwich.

The welding electrodes were grinded using a diamond grinder plate and sand paper until a round edge at the tip is obtained that could be precisely pressed on top of the sandwich. The quality of the welds was very dependent on the pressure applied to each of the penholder-like handles.

The welds were made using the bipolar technique shown in Fig. 2c, where the electrical current flowed from the positive electrode through two platinum strips, through all the stainless-steel wires and through the copper base. It was clear that below the positive electrode special attention had to be focused on the applied pressure. With respect to this, the quality of the weld can of course differ depending on the internal resistance that the operator happens to achieve during the preparation. Thus, the obtained welded interconnection between the stimulating electrode and lead wire with the indicated cross-section A-A where the metallographic analysis was performed is shown in Fig. 2b.

The resistance of a lead wire welded to a single stimulating electrode was 39 Ω. In the quasi-biphasic stimulating connection, however, the total resistance of the lead wires attached to a specific triplet of the stimulating electrodes was 58 Ω.

Finally, the sheet containing the matrix of thirty-three electrodes was then adhered to the inner side of the opened cuff.

### Metallurgical samples

Metallographic analysis was used to analyse any failure and determine the microstructure of the weld in order to set up the optimum welding conditions. To reveal the microstructure, the method of scanning electron microscopy was used. For the metallographic analysis a FEG SEM JEOL 6500 F field-emission scanning electron microscope with energy-dispersive spectroscopy (an INCA X-SIGHT LH2-type detector, INCA ENERGY 450 software) was used.

The sample for the microstructure observation was taken at the cross-section A-A of the welded interconnection between the stimulating electrode and the lead wire, as indicated in Fig. 2b. The samples were prepared according to the classic metallographic procedure (sectioning, embedding the strip samples in resin, grinding and polishing). They were placed in resin using special holders to ensure the specimen’s position for further analysis. The samples prepared for the scanning electron microscope analysis were grinded and polished without etching.

### Mechanical tests

Destructive load tests of the welded interconnection were performed with an INSTRON servohydraulic testing machine (Model: 8802, 250 kN, Norwood, MA, U.S.A.) intended for static and dynamic testing up to 25 or 250 kN, frequencies up to 80 Hz and temperatures up to 1250 °C. For this purpose six samples of electrodes with interconnected lead wires were made. Afterwards, each of them was attached with the free end to the measuring head and the end with the electrode and interconnection was attached to the special holder. Then the displacement between the ends was enlarged until breakage of the interconnection occurred, while the load was measured continuously.

The mechanical properties of the aforementioned weld sample were measured at eight sites of the weld sample using the Fischerscope Vickers nanohardness tester (Type: H100C, Helmut Fischer, Sindelfingen-Maichingen, Germany) with the working load range: 0.4 mN to 1 N. The measuring force used was 30 mN.

### Tests of functionality

Tests of the functionality of the cuff were performed using measurements of the threshold and the selectivity of the stimulation in an isolated porcine vagus nerve that was assessed while using a specific current, biphasic quasitrapezoidal pulses^51,52^. They were applied to the nerve via a specific group of three electrodes. To assess which nerve fibres made the most contribution to the compound action potential (CAP) during the selective vagus nerve stimulation, three components of interest, i.e., the maximum CAP deflection, the latency of the maximum CAP deflection and the conduction velocity, were identified.

## Results

**Figure 3:**
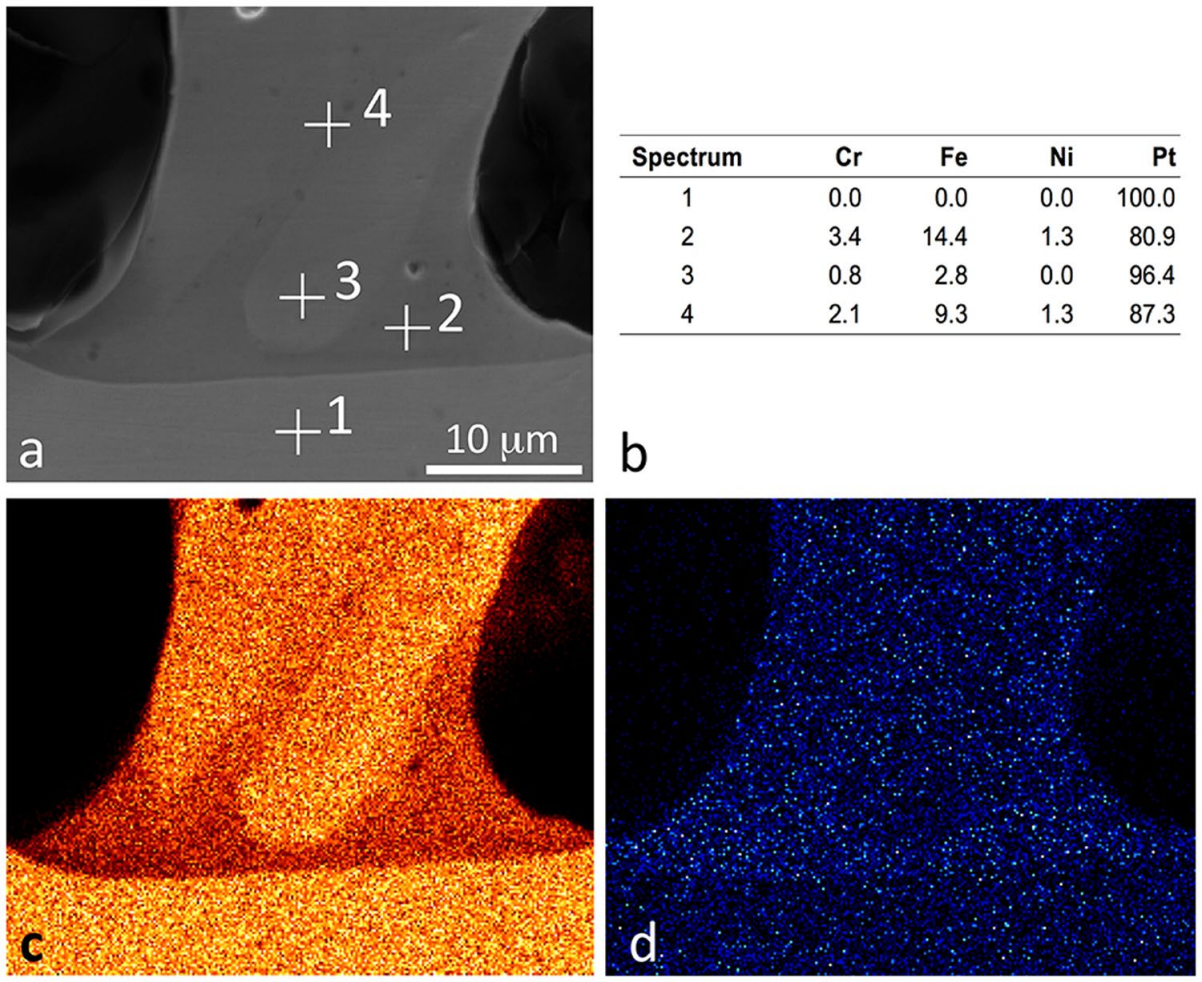
BEI of the welded AISI 316 L wire and Pt foil with four sites of interest at the weld sample (**a**) where the elements Cr, Fe, Ni and Pt are analysed in weight percent (**b**), according X-ray images Fe Kα (**c**) and Pt Mα (**d**) are also given.

The energy stored for a charging current of 5 A and a charging voltage of 3 V resulting in a stored energy of 13.5 W (see Fig. 2c) was the optimum welding energy that produced a reliable weld. At these values, most of the stainless-steel leading wires appeared molten and it was concluded that alloying had occurred. This could be attributed to the significant difference of approximately 380 °C between the melting points and the electrical resistivity of the two welded materials. This is clearly confirmed by the results of the scanning electron microscopy analysis for the welded interconnection between the stimulating electrode and the lead wire. Figure 3 shows a magnified part of the weld between the stainless-steel wire and the platinum foil. The microscopic observation showed that the weld is sound and does not exhibit any typical welding defects, such as oxide films, oxide inclusions, gas bubbles or shrinkage porosity. In fact, the images in Fig. 3 represent the area where the partial melting of the stainless-steel wire occurred. The melting of the steel wire caused the partial dissolution of the platinum foil into the weld melt. The Pt Mα X-ray signal (orange) shows an increased concentration of platinum in the weld melt. Due to the differences in atomic mass the partial “alloying” of the stainless-steel weld melt can be observed on the backscattered electron image (BEI) as lighter colours in the dark-grey wire. The partially melted stainless-steel wire also shows signs of a small wetting angle with the Pt substrate. This means that the likelihood of good weld binding is increased.

The images in Fig. 3 show a stainless-steel wire that was melted during welding. The mixing of the platinum foil and the stainless-steel wire was significant. Most of the wire was infiltrated by the Pt melt. Interestingly, while all of the steel wire now contains some Pt, there is still a distinct border between the Pt foil and the Fe-based weld on one side, while on the other side of the weld, the Pt melted from the foil and mixed with the steel weld melt. The image in Fig. 3a shows electron-microscope images of the sites of interest in the weld sample where the elements Cr, Fe, Ni and Pt were analysed. Figure 3a and b also show the points on the electron images and the content of the elements expressed in weight percent.

**Figure 4:**
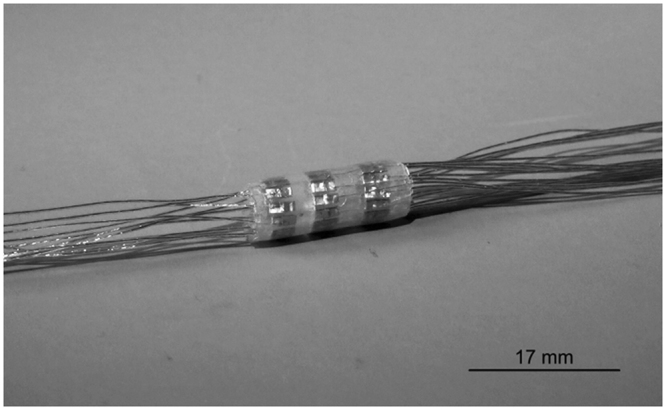
Manufactured stimulating spiral nerve cuff.

The manufactured stimulating spiral nerve cuff sample is given in Fig. 4. The sample contains welds that are manufactured in the same way as the weld that was analysed in Fig. 3.

### Tests of functionality

Destructive load tests performed on six samples of electrodes with interconnected lead wires made using the servohydraulic testing machine show that the breaking loads are within the range 3.22 to 5 N.

The mechanical properties of the weld sample performed at eight sites of the weld using the nanohardness tester showed that the hardness was dependent on a particular site into the weld: the hardness of the platinum foil was 112 HV, while the hardness of unaffected 316 L wire was 263 HV. Furthermore, the weld hardness was 218 HV in the middle of the melted wire, 275 HV in the area of stainless steel and Pt mixing, 164 HV on the Pt/stainless-steel wire interface, and 108 HV in the foil just below the weld.

The results show that for different stimulation intensities (i_c_) both the CAP deflection and the shape are influenced. In particular, at high i_c_, the latency was low and the corresponding conduction velocity was high for all. At low i_c_, however, the latency was high and the corresponding conduction velocity was low. It can be assumed that at low i_c_, large Aβ fibres having low threshold are recruited first. As the i_c_ increased, however, the next recruited are the fast B fibres. The measured CAPs did not show separate peaks corresponding to the A- and B-fibre types.

**Figure 5:**
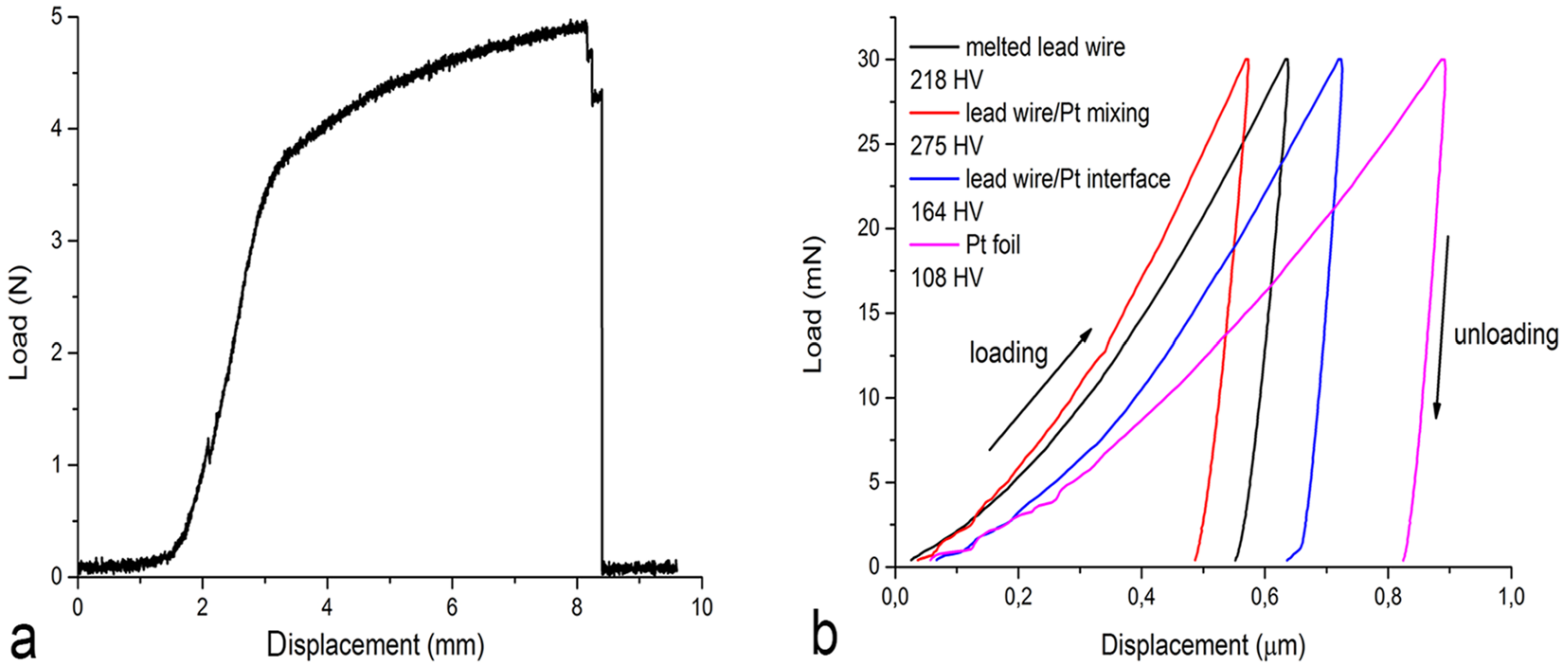
Measurements of mechanical properties (**a**) destructive tensile test, (**b**) weld nanohardnes tests.

**Figure 6:**
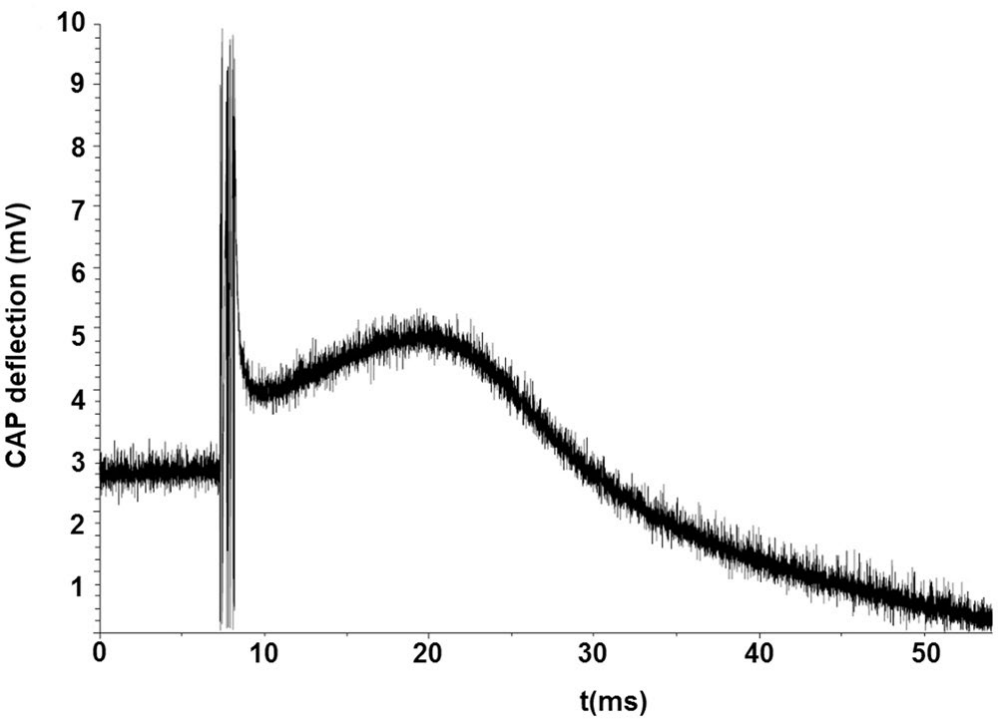
Measurements of compound action potential (CAP).

In Fig. 5, examples of the alterations measured in the mechanical tests and the test of the functionality are shown. In particular, Fig. 5a shows the change in the load applied during the destructive load test in the sample electrode with interconnected lead wire number 6 until breakage. Furthermore, Fig. 5b shows the upload and download trajectories at eight sites on the weld sample applied by the nanohardness tester. Figure 6 shows the change of the CAP recorded at an i_c_ of 7 mA with a particular longitudinal group of three electrodes number 6, also comprising a stimulus artefact.

## Discussion

The study is focused on improving the methods developed for the construction and crafting of a multi-electrode spiral stimulating nerve cuff capable of the selective activation of specific superficial regions of a nerve trunk as well as a certain degree of fibre-type selective stimulation. In particular, the purpose of the study was to test the hypothesis that spot welding, using a standard single pulse applied under optimum welding conditions, is a feasible technique for interconnections between platinum stimulating electrodes and stranded stainless-steel lead wires within the cuff. As a result, a thirty-three-electrode stimulation cuff for the selective activation of different superficial compartments, particularly within the vagus nerve, was manufactured.

In recent decades cuffs have been built that cover the size range from 2 to 5 mm ID and 14 mm length, with the number of active triplets from five to eleven^5^. In general, most nerve cuffs are manufactured using medical-grade silicone materials in combination with platinum foils that are connected to stainless-steel wires using different technologies. Nevertheless, there is still a lack of detailed metallurgical and electrochemical analyses regarding the technologies of interconnection. Therefore, the possibility of lead failure during prolonged nerve stimulation imposes some constraints on the use of these techniques for clinical applications.

Many different concepts for peripheral nerve interfaces have been developed and transferred into research or clinical applications with varying degrees of success. The major advantages of the technology deployed in the crafting are enhanced reliability, well-known body reactions, and the softness of the silicone sheets. In this relation, advances in vagus nerve stimulation have increased the need for nerve cuff designs that can selectively modulate the function of an internal organ via the selective stimulation of selected populations of nerve fibres^9,18^. Accordingly, the spiral-like arrangement of the cuff placed around the vagus nerve potentially allows an appropriately tight cuff-to-nerve contact. At the same time, the hypothesis that spiral cuff electrodes can be implanted with an internal diameter less than that of the nerve and expand to accommodate the vagus nerve without compression can be accepted. Therefore, with the goal of minimizing problems related to the cuffs and even improve their effectiveness, the technology of crafting on the basis of theoretical models as well as the appropriate technological solutions should be used.

Like with the majority of cuffs, the major disadvantages of the fabricated cuff are a relatively large cover of the nerve trunk, preventing natural circulation of the metabolites, rigidity and radial pressure. The more channels it has the more rigid is the whole cuff. Therefore, in crafting the cuff, the compressive action of the cuff and the potential chronic neuropathy possibly induced by the cuff should be carefully considered. In addition, the changed mechanical environment and the physiological movements of the tissue created by the presence of a cuff could have significant and acute effects on both the cuff’s performance and the nerve’s function^64,65^. Namely, responses to compressive effects could include axonal degeneration, demyelination, fibrosis, and are sometimes associated with a loss of electrophysiological function^66^. According to previous *in-vivo* testing of a cuff, the authors assumed that the pressures exerted by the developed cuff generate only a small amount of pressure, which is below the pressure required to occlude the blood flow in the nerves^18,30,39^.

One, very important, disadvantage is that the spatial selectivity strongly depends on the position of the electrodes relative to the nerve and its inner fascicles. It appears to be nearly impossible to selectively excite only the targeted few fibres in compound fascicles of, *e.g*., a vagus nerve using a cuff. Beside this, it was shown by Grill and Mortimer^36^ that the resistivity of the encapsulation tissue that surrounds cuffs chronically implanted on the sciatic nerve in adult cats for up to 8 months is sufficient to alter the shape and magnitude of the electric field generated by the chronically implanted electrodes. Furthermore, in certain circumstances, significant chemical reactions can occur during the electrical stimulation that can cause potentially destructive electrochemical effects on the welded interconnection between the platinum electrode and the stainless-steel lead wire. It was shown in the recent study of Pečlin *et al*.^67^, reviewing the electrochemical performance of a platinum electrode within a multi-electrode spiral nerve cuff, that the power spectral density of a quasi-trapezoidal, biphasic and current stimulating pulse, exhibits two main peaks, one at about 1 kHz and the other at about 2.5 kHz. The corresponding impedances for the two peaks are 600 Ω and 450 Ω, respectively. Therefore, the common resistance of wires leading to the single electrode triplet in the quasi-biphasic stimulating connection of 58 Ω is relatively low compared to the resistance of being in contact with the nervous tissue.

Nevertheless, the disadvantages are also the costs of their crafting, i.e., the cuffs are handcrafted and the costs rise in proportion to the number of stimulation electrodes due to the additional fabrication effort.

According to most of these constraints, the results of the present study are consistent with the published results of other investigators^5,9,18,39^. However, further research that would be necessary to answer the questions raised by the results could proceed in the following directions:

- “*in-vitro*” flex bending fatigue testing to develop fatigue-life curves of the interconnections between the lead wires and the stimulating electrodes,
- topological *in-vitro* assessments of the superficial properties of the welds using the electrochemical method of cyclic voltammetry instrumented with the reference electrode having a fine outside tip (diameter of 10 µm),
- cuff designs to reduce the mechanical perturbations via the utilization of materials more closely suited to the mechanical properties of the nerve tissue and thus reduce the mechanical charge of the welded interconnections,
- *in-vivo* studies to directly correlate acute function with chronic neural structure and function,
- performing more experiments to show group statistics.

In conclusion, our findings emphasize the importance of mechanically reliable and electrochemically safe interconnections between a platinum stimulating electrode and a stainless-steel lead wire obtained using spot welding. The work presented has implications for nerve cuff design, implantation, and the prediction of long-term efficacy. It is imperative to understand all the problems related to the materials that may occur when a cuff is used for nerve stimulation over longer periods of time, which can sometimes be as long as 20 years.

We believe that the present study has contributed to the further development of cuffs to be used for the efficient and safe selective stimulation of autonomous peripheral nerves and for the selective recording of neural responses at the same time.
