# Publisher Correction: An improved method of crafting a multi-electrode spiral cuff for the selective stimulation of peripheral nerves

**DOI:** 10.1038/s41598-018-35697-6

**Published:** 2018-11-19

**Authors:** Janez Rozman, Polona Pečlin, Samo Ribarič, Matjaž Godec, Jaka Burja

**Affiliations:** 1 Center for Implantable Technology and Sensors, ITIS d. o. o. Ljubljana, Lepi pot 11, 1000 Ljubljana, Slovenia; 2 Institute of Pathophysiology, Medical Faculty, University of Ljubljana, Vrazov trg 2, 1000 Ljubljana, Slovenia; 3 Institute of Metals and Technology, Lepi pot 11, 1000 Ljubljana, Slovenia

Correction to: *Scientific Reports* 10.1038/s41598-018-19318-w, published online 17 January 2018

The original version of this Article contained an error in the title of the paper, where the phrase “stimulation of peripheral nerves” was omitted. This has now been corrected in the PDF and HTML versions of the Article.

